# Awareness, Concerns, and Protection Strategies Against Bloodborne Viruses Among Surgeons

**DOI:** 10.7759/cureus.4242

**Published:** 2019-03-12

**Authors:** Anadel Hakeem, Shahad Alsaigh, Amal Alasmari, Amairah Aloushan, Fatemah Bin Saleh, Zeyad Yousef

**Affiliations:** 1 Internal Medicine, King Saud Bin Abdulaziz University for Health Sciences, College of Medicine, Riyadh, SAU; 2 Surgery, King Saud Bin Abdulaziz University for Health Sciences, College of Medicine, Riyadh, SAU; 3 Dermatology, King Saud Bin Abdulaziz University for Health Sciences, College of Medicine, Riyadh, SAU; 4 Emergency Medicine, King Saud Bin Abdulaziz University for Health Sciences, College of Medicine, Riyadh, SAU; 5 Pediatrics, King Saud Bin Abdulaziz University for Health Sciences, College of Medicine, Riyadh, SAU

**Keywords:** surgeon, needlestick injury, hiv, hbv, hcv

## Abstract

Background: Surgeons are at high risk of contracting infectious viruses such as human immunodeficiency virus (HIV), hepatitis B virus (HBV), and hepatitis C virus (HCV) through exposure to patients’ blood. The purpose of this study was to assess the surgeons’ awareness of contracting bloodborne viruses.

Methods: A cross-sectional study with a questionnaire distributed to 241 surgeons at King Abdulaziz Medical City - Riyadh (KAMC-R) during the period June 2017 through January 2018. Descriptive statistics were used to analyze data collected using Stata®, v14 (StataCorp LLC, College Station, Texas, USA). Categorical variables were analyzed using Pearson chi-square test. P-value of < 0.05 was considered significant.

Results: A total of 241 surgeons answered the questionnaire, 179 (74.3%) surgeons were male and 62 (25.7%) were female. The mean age ± standard deviation (SD) of male surgeons was 35.8 ± 11.0 years while for females was 33.3 ± 9.1 years. The majority of our cohort were vaccinated for HBV (96% in males and 97% in females). Two-thirds of the study cohort did not know the conversion rate post-needlestick injury by HIV, HBV, and HCV. Two-thirds of the study cohort think there is a need for HIV screening before surgery. Mixed answers were received from the cohort when asked about their concern regarding contracting HIV infection from their patients; only one-third of the surgeons were extremely concerned. When asked about the risk of needlestick injury during treating patients positive for HBV, the majority of the surgeons said no. However, a significant difference between the female and male surgeons was found in which 12 of the 62 female surgeons answered yes (19.4%) compared to 11 of the 179 male surgeons (6.1%) (p = 0.002).

Conclusion: The majority of our surgeons are vaccinated for HBV. However, female surgeons appear to be at higher risk of needlestick injury from HBV patients. This requires further investigation into the reasons for such high incidents. More education is needed about bloodborne viruses.

## Introduction

Surgeons are at high risk of contracting infectious viruses, such as the human immunodeficiency virus (HIV), hepatitis B virus (HBV), and hepatitis C virus (HCV), through exposure to patients’ blood [1-5]. The prevalence of HBV in the Kingdom of Saudi Arabia (KSA) decreased significantly after the introduction of the HBV vaccine; it dropped from 7% to 0.3% [6]. According to the World Health Organization (WHO), the reported prevalence of HCV in KSA is 1.8%, and the HIV annual incidence in KSA is less than 4/100,000 [7-8]. The risk of infection after an occupational exposure for healthcare workers (HCWs) to HBV (unvaccinated), HCV, and HIV is between 6% to 30%, 1.8%, and 0.3%, respectively [3]. However, the cumulative lifetime risk increases dramatically with increased years of experience for surgeons and repetitive exposure to blood [9-11]. Mckinney et al. reported that the cumulative risk was 10% for the surgeon operating on HIV-positive patients for more than a 30-year profession [12]. In addition, a national survey conducted in Italy demonstrated that the 30-year lifetime risk of getting an HBV, HCV, or HIV infection for surgeons was 42.7%, 34.8%, and 0.54%, respectively [13]. A study was done in Najran in 2014 to evaluate seroprevalence of HBV and HCV among medical students and HCWs which indicated a seroprevalence of HBV of 1.7% and 8.7% for medical students and HCWs, respectively [14]. In addition, the seroprevalence of HCV was 0% and 0.3% in medical students and HCWs, respectively.

Needlestick injuries are one of the occupational risks for HCWs, and it is a route for transmitting bloodborne diseases [2, 4, 15]. A study conducted on the Ministry of Health Hospitals of Saudi Arabia estimated that the annual incidence of percutaneous injuries was 3.2 per 100 occupied beds [16]. El-Hazmi et al. reported that 14.3% of needlestick injuries happened during surgery at King Khalid University Hospital, Riyadh [17]. However, most of the needlestick injuries are underreported by HCWs worldwide and in Saudi Arabia [15, 18-23]. A study done by Patterson et al. showed that only 17% of surgeons reported needlestick injuries [18]. In addition, a study by Samargandy et al. was done in Jeddah to evaluate the clinical consequences of occupational exposure to blood and other body fluids which revealed surgeons had a higher risk of needlestick injuries in comparison to other doctors [24]. After reviewing 326 charts of occupational exposure, all of the exposed HCWs did not seroconvert to HIV, HBV, or HCV with adequate post-exposure prophylaxis.

To the limit of our knowledge, no study has been conducted in Saudi Arabia to evaluate surgeons’ awareness of infectious viruses in the operating room and their reporting of needlestick injuries. Therefore, the purpose of this study was to assess the surgeons’ awareness of contracting bloodborne viruses. 

## Materials and methods

Study area/setting

This study was performed at the Department of the Surgery, Obstetrics/Gynecology, and Cardiac Center at King Abdulaziz Medical City, Riyadh (KAMC-R), Saudi Arabia. A questionnaire was completed by surgeons from different subspecialties.

Study subjects

This study included all surgeons from different positions who were working at KAMC-R. Different surgical subspecialties were included (Cardiovascular, Vascular, General Surgery, Neurosurgery, Ophthalmology, Plastic, Otolaryngology, Urology, Orthopedics, Thoracic, and Obstetrics/Gynecology) as were different surgical positions, including residents, associate consultants, fellows, and consultants. Oral and maxillofacial surgeons and other healthcare workers, including nurses, medical students, and interns, were excluded.

Study design

This was a cross-sectional study with a questionnaire distributed to 241 surgeons at KAMC-R during the period of June 2017 to January 2018.

Sample size

The sample size was calculated using the Roasoft online calculator (www.raosoft.com/samplesize.html) assuming a 5% margin of error, 95% confidence level, the population size (the number of surgical staff at KAMC-R was 318), and a 50% response distribution. The necessary sample size was 175.

Data collection methods, instruments used, measurements

The questionnaire was sent by email to surgeons. Due to an inadequate response, personal interviews were then conducted. The questionnaire was delivered in an electronic Google form, as well as a hard copy, based on the surgeons' preferences. The questionnaire was adapted and modified from two previous studies by Wright and Patterson [18, 25]. The questionnaire included demographic information, such as age, gender, subspecialty, and surgical experience. It also addressed the risk of transmission, awareness of seroconversion rates, and reporting patterns of needlestick injuries.

Data management and analysis plan

Descriptive statistics were used to analyze the data collected using Stata software v14 (StataCorp LLC, College Station, Texas, USA). Categorical variables were analyzed using the Pearson chi-square test. Risk of needlestick injury was measured by the odds ratio (OR) and 95% confidence interval (CI). A P-value of < 0.05 was considered significant. This research was also presented in 2018 (Abstract: Hakeem A, Alsaigh S, Alasmari A, Aloushan A, Bin Saleh F, Yousef Z: Awareness, concerns and protection strategies against bloodborne viruses among surgeons. Patient Safety Forum, Riyadh, Saudi Arabia, April 9, 2018).

## Results

A total of 241 surgeons answered the questionnaire; 179 (74.3%) surgeons were males and 62 (25.7%) were females. The mean age ± standard deviation (SD) of male surgeons was 35.8 ± 11.0, while for females, it was 33.3 ± 9.1. The female mean surgical experience ± SD was 7.8 ± 7.8 and for males was 8.6 ± 8.8. A summary of the baseline surgeons' demographics is outlined in Table 1. The majority of surgeons were vaccinated against HBV (96% in males and 97% in females) with no statistically significant difference between genders (p = 0.672) (Table 2).

**Table 1:** Demographic Characteristics of Surgeons SD: standard deviation; yrs: years

Sex	Male	Female
N (%)	179 (74.3)	62 (25.7)
Mean age (yrs) ± SD	35.8 ± 11.0	33.3 ± 9.1
Specialty N (%)		
Cardiovascular surgery	6 (3.4)	0 (0)
Neurosurgery	14 (7.8)	3 (4.8)
Obstetrics/Gynecology	22 (12.3)	34 (54.8)
Pediatric surgery	8 (4.5)	3 (4.8)
General surgery	44 (24.6)	10 (16.1)
Orthopedic surgery	23 (12.8)	0 (0)
Plastic surgery	4 (2.2)	1 (1.6)
Otolaryngology	19 (10.6)	5 (8.1)
Urology	21 (11.7)	0 (0)
Thoracic surgery	0 (0)	1 (1.6)
Vascular surgery	4 (2.2)	0 (0)
Ophthalmology	14 (7.8)	5 (8.1)
Surgical position N (%)		
Consultant	43 (24)	11 (17.7)
Associate consultant	14 (7.8)	5 (8.1)
Assistant consultant	14 (7.8)	3 (4.8)
Fellow	6 (3.4)	4 (6.5)
Resident	98 (54.7)	38 (61.3)
Staff physician	5 (2.8)	1 (1.6)
Mean experience (yrs) ± SD	8.6 ± 8.8	7.8 ± 7.8

**Table 2:** Number and Percentage of Surgeons Who Had Been Vaccinated Against Hepatitis B

	Male N (%)	Female N (%)	P-value
Are you vaccinated against hepatitis B?			
Yes	171 (95.5)	60 (96.8)	0.67
No	8 (4.5)	2 (3.2)	

Surgeons’ awareness of serum conversion rates of bloodborne viruses

Almost two-thirds of the surgeons (male and female) were unaware of the conversion rate post-needlestick injury by HIV, HBV, and HCV (Table 3).

**Table 3:** Surgeons’ Knowledge of the Conversion Rate for Bloodborne Viruses After Needlestick Injury HBV: hepatitis B virus; HCV: hepatitis C virus; HIV: human immunodeficiency virus

	Male N (%)	Female N (%)	P-value
Conversion rate for HCV after needlestick			
Correct answer	56 (31.3)	18 (29.0)	0.59
Wrong answer	123 (68.7)	44 (70.9)	
Conversion rate for HBV after needlestick			
Correct answer	69 (38.6)	20 (32.3)	0.21
Wrong answer	110 (61.4)	42 (67.7)	
Conversion rate for HIV after needlestick			
Correct answer	62 (34.6)	18 (29.0)	0.68
Wrong answer	117 (65.4)	44 (70.9)	

HIV concerns

Almost two-thirds of the surgeons believed that there is a need for HIV screening before surgery (Table 4). Mixed answers were given about their concern regarding contracting HIV from patients; only one-third of the surgeons were extremely concerned about contracting HIV (Table 4).

**Table 4:** Reported Surgeons' Beliefs About HIV Screening and Their Concerns About Contracting HIV HIV: human immunodeficiency virus

	Male N (%)	Female N (%)	P-value
Do you think that patients must be regularly screened for HIV before surgery?			
Yes	108 (60.3)	44 (70.9)	0.14
No	71 (39.7)	18 (29.0)	
How concerned are you about HIV transmission through your work?			
Extremely	62 (34.64)	23 (37.1)	0.70
Very	25 (14.0)	9 (14.5)	
Moderately	35 (19.6)	11 (17.7)	
Slightly	42 (23.5)	17 (27.4)	
No concern	15 (8.4)	2 (3.2)	

Reporting needlestick injuries

Almost half of the surgeons (115, 47.7%) rarely or never reported a needlestick injury (Table 5). Table 6 shows the self-reported occurrence of needlestick injuries by male and female surgeons. Female surgeons were at a higher risk of needlestick injury while treating patients with HIV, HBV, and HCV infections. However, the only statistical significance was noticed in female surgeons treating an HBV positive patient (OR 3.7, 95% CI: 1.4 - 9.7, p = 0.002).

**Table 5:** Frequency of Reporting Needlestick Injury Among Surgeons

	N (%)
How frequently do you report a needlestick injury?	
Always	77 (31.9)
Sometimes	13 (5.4)
Occasionally	36 (14.9)
Rarely	54 (22.4)
Never	61 (25.3)

**Table 6:** Number and Percentage of Surgeons Who Had Been Stuck by a Needle from Patients with Bloodborne Viruses with Odds Ratio and 95% Confidence Intervals ^a ^Reference Group CI: confidence interval; HBV: hepatitis B virus; HCV: hepatitis C virus; HIV: human immunodeficiency virus; OR: odds ratio

		N (%)	OR (95% CI)	P-value
HIV	Male	2 (1.2)	Ref^a^	0.076
	Female	3 (5.1)	4.5 (0.5 - 54.7)	
HBV	Male	11 (6.2)	Ref^a^	0.002
	Female	12 (19.4)	3.7 (1.4 - 9.7)	
HCV	Male	12 (6.7)	Ref^a^	0.127
	Female	8 (12.9)	2.1 (0.7 - 5.8)	

## Discussion

The aim of this study was to investigate the awareness of surgeons about bloodborne diseases. Most of our cohort of surgeons were vaccinated against HBV; however, a small proportion still remains unvaccinated. The majority of the surgeons in this study were not aware of the conversion rates of the bloodborne pathogens after needlestick injury. Female surgeons were also at higher risk of needlestick injury when treating patients with HIV, HBV, or HCV.

As evidenced by previous studies, surgeons are at increased risk of contracting bloodborne viruses [1-5, 9-13]. Hepatitis B vaccination is recommended for all healthcare workers by the Center for Disease Control (CDC) [4]. However, local and international studies have reported low adherence to HBV vaccination among HCWs [14, 26-29]. In contrast, 95% of surgeons were vaccinated in this study. Although surgeons were extremely concerned regarding contracting bloodborne viruses, only a small number reported a needlestick injury which is similar to previous studies [15, 17-23]. A lack of awareness was common among surgeons regarding the seroconversion rate as the majority of them answered the questionnaire incorrectly. Therefore, surgeons need to be more educated about the risk of transmission of bloodborne pathogens and ways of protection. Reporting needlestick injuries was not a widespread practice among the surgeons in this study, which is alarming. This has also been noted in other studies [15, 17-23]. In this survey, the average number of needlestick injuries per year was similar to other studies.

An interesting finding of this study was that female surgeons were more likely to be stuck by a needle while treating a patient positive for HBV, which necessitates the importance of education about needlestick injury and their risk. Why females are at higher risk of needlestick injury was not addressed in this survey; however, it could be that male surgeons are underreporting needlestick injuries. On the other hand, 53.8% of HCWs did not report needlestick injuries in a governmental hospital in Medina, Saudi Arabia [19]. Even in the United Kingdom (UK), under-reporting of needlestick injuries is a widespread practice among surgeons, according to a study where only 2.26% of injuries were reported to occupational health [20]. Junior surgeons had a higher probability to report needlestick injuries versus senior surgeons (9.82% vs. 1.10%) [20]. Since most of the HCWs do not report their needlestick injuries, they will not get their post-exposure prophylaxis, which leads to an increase in the risk of acquiring bloodborne diseases [2]. A high percentage of surgeons believe that patients should be screened for HIV, even though it is not prevalent in Saudi Arabia [8].

Based on the study findings, surgeons must implement protection strategies against bloodborne pathogens and should be obliged to do so. Also, more education to surgeons regarding the needlestick injuries protocol is required as some do not have the knowledge on how to report it. One of the study limitations was the surgeons’ busy schedules; this influenced their ability to answer the questions precisely as they wanted to finish quickly which affects the accuracy of the responses. In addition, the study had a small sample size compared to similar studies. Therefore, a national survey is recommended to draw consistent conclusions.

## Conclusions

In conclusion, reporting needlestick injuries is not a widespread practice among surgeons, which may lead to an increased risk of contracting bloodborne infections and less use of prophylaxis as mandated by the CDC and WHO. In addition, female surgeons appear to be at a higher risk of needlestick injury. This requires further investigation into the reasons for such high incidents.
